# Primary versus recurrent surgery for glioblastoma—a prospective cohort study

**DOI:** 10.1007/s00701-020-04605-1

**Published:** 2020-10-14

**Authors:** Maja Chava Rubin, Lisa Millgård Sagberg, Asgeir Store Jakola, Ole Solheim

**Affiliations:** 1 Department of Neuromedicine and Movement Science, Faculty of Medicine and Health Sciences, NTNU, Norwegian University of Science and Technology, Trondheim, N-7491 Norway; 2 Department of Neurosurgery, St. Olav’s University Hospital, Trondheim, Norway; 3 Department of Neurosurgery, Sahlgrenska University Hospital, Gothenburg, Sweden; 4 Institute of Neuroscience and Physiology, Sahlgrenska Academy, Gothenburg, Sweden

**Keywords:** Glioblastoma, Neurosurgery, Patient-reported outcome measures, Quality of life

## Abstract

**Background:**

There is currently limited evidence for surgery in recurrent glioblastoma (GBM). Our aim was to compare primary and recurrent surgeries, regarding changes in perioperative, generic health-related quality of life (HRQoL), complications, extents of resection and survival.

**Methods:**

Between 2007 and 2018, 65 recurrent and 160 primary GBM resections were prospectively enrolled. HRQoL was recorded with EQ-5D 3L preoperatively and at 1 month postoperatively. Median perioperative change in HRQoL and change greater than the minimal clinically important difference (MCID) were assessed. Tumour volume and extent of resection were obtained from pre- and postoperative MRI scans. Survival was assessed from date of surgery.

**Results:**

Comparing recurrent surgeries and primary resections, most variables were balanced at baseline, but median age (59 vs. 62, *p* = 0.005) and median preoperative tumour volume (14.9 vs. 25.3 ml, *p* = 0.001) were lower in recurrent surgeries. There were no statistically significant differences regarding complication rates, neurological deficits, extents of resection or EQ-5D 3L index values at baseline and at follow-up. Twenty (36.4%) recurrent resections vs. 39 (27.5%) primary resections reported clinically significant deterioration in HRQoL at follow-up. Stratified by clinically significant change in EQ-5D 3L, the survival distributions were not statistically significantly different in either group. Survival was associated with extent of resection (*p* = 0.015) in recurrent surgeries only.

**Conclusions:**

Outcomes after primary and recurrent surgeries were quite similar in our practice. As surgery may prolong life in patients where gross total resection is obtainable with reasonable risk, the indication for surgery in GBM should perhaps not differ that much in primary and recurrent resections.

## Introduction

Glioblastoma (GBM) is the most common primary malignant brain tumour in adults [24]. Newly diagnosed GBM is usually treated with so-called maximal safe surgical resection if feasible, followed by adjuvant radiation and concomitant and often adjuvant chemotherapy [34]. Despite multimodal treatment, relapse is inevitable, and the prognosis remains poor. However, due to the lack of evidence, there is no consensus on the best way to treat recurrent GBM. It is so far not settled whether reoperations for GBM prolong survival [9, 40]. Neither is the literature conclusive concerning the potential dose-response relationship of extent of resection on survival after recurrence [2, 6, 18, 23, 25, 26, 28, 29, 32, 35, 37, 39]. Hence, some hospitals never offer resections at recurrence, although others do.

Some studies report increased rates of surgery-related morbidity and complications in recurrent disease [6, 21, 29], and potential survival benefit should be balanced against other clinical outcomes [20]. While several studies mention health-related quality of life (HRQoL) as an essential aspect in this palliative setting, patient-reported perioperative HRQoL is barely studied in this context [3, 9, 10, 17, 27, 36].

In the present prospective cohort study, we sought to compare treatment results after primary versus recurrent surgeries for GBM, in terms of changes in perioperative and generic HRQoL, complication rates, extents of surgical resection and survival.

## Material and methods

### Study population

**Fig. 1:**
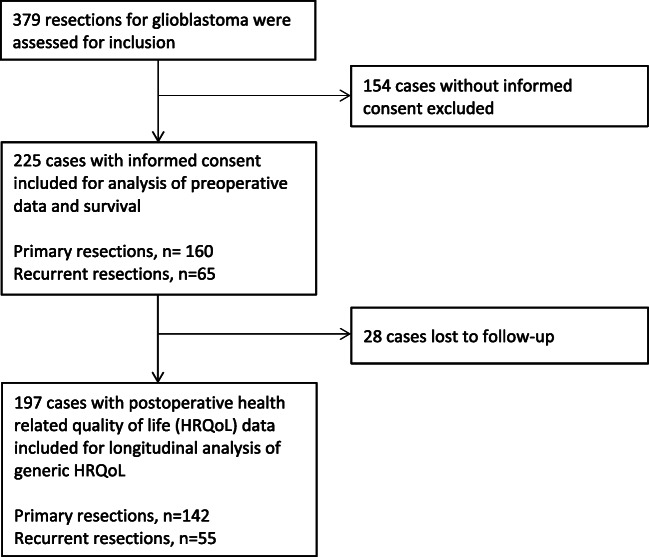
Flow chart showing the inclusion process

The project is based on prospectively collected data from patients ≥ 18 years of age, undergoing surgery for GBMs at St. Olavs University Hospital, Norway, from September 2007 through August 2018. Patients eligible for enrolment were patients considered suited for surgery based on local treatment indications. Patients were included after informed consent. At St. Olavs Hospital, recurrent surgery is often considered (although with exemptions) an option for patients who are functionally independent (e.g. Karnofsky performance status (KPS) ≥ 70), enhancing tumour volumes believed available for gross total resection, and when recurrence takes place at least six months after primary resections. The histopathological diagnosis of GBM was graded and confirmed by a neuropathologist, according to the World Health Organization (WHO) classification at time of diagnosis [13, 16]. Patients with histopathological anaplastic astrocytoma and evidence of necrosis on magnetic resonance imaging (MRI) were also included, as they are shown to have the same prognosis as patients with histopathological grade IV astrocytoma [15] and were treated as GBMs at our hospital. Patients with prior histopathological diagnosis of glioma grade I–III without evidence of necrosis on MRI, who transformed to GBM at a later stage, were not included. Multifocal tumours operated in more than one session were assessed as single operations. There were 65 recurrent surgeries included in the study, 19 of which were conducted on eight patients. Twenty-eight patients underwent both primary and recurrent resections and were included in both groups. The inclusion process is shown in the flowchart labelled Fig. 1. Amongst the 23 patients still alive at the end of follow-up, the median (range) duration of follow-up was 23 months (10–96).

### Collection of data

A HRQoL-questionnaire was completed one to three days preoperatively by self-administration or with assistance from next of kin or a nurse, and at one month postoperatively in a structured telephone interview conducted by a research nurse. KPS was scored by the operating surgeon just prior to surgery and by a research nurse in a structured phone interview one month postoperatively. KPS was missing in 10 patients and was therefore assessed retrospectively from electronic medical records and dichotomized to above and below 70. Charlson Comorbidity Index (CCI) [4], postoperative complications within 30 days postoperatively classified with the Landriel classification [14] and new or worsened neurological deficits at the time of discharge were collected retrospectively from electronic medical records from the university hospital and the seven local hospitals in the catchment region.

Tumour volume data was obtained from pre- and early postoperative MRI scans (taken within 72 h of surgery) and calculated with the ellipsoid formula ((4*πr*_1_ · *r*_2_ · *r*_3_)/3, where *r*_*x*_ was defined as diameter/2 from the largest perpendicular diameters in perpendicular image dimensions). In 28 selected cases with oddly shaped tumours, volume data was obtained by semi-automatic tumour segmentation (3D slicer). Only contrast-enhancing tumour tissue and tissue within the enhancement rim was included when calculating the pre- and postoperative volumes. For recurrent tumours, tumour cavities were subtracted from the tumour volume if there was a clear fluid signal on fluid-attenuated inversion recovery (FLAIR) images. In three patients, MRI images were not available, and computed tomography (CT) images were used in tumour volume assessments. The extent of resection was classified as either gross total (100%), near total (90–99%) or subtotal resection (< 90%). Eloquence was graded as suggested by Sawaya et al. [31], but we chose to grade hippocampal tumours as eloquent as well. For multifocal tumours, the most eloquent part of the tumour was scored.

### HRQoL instrument

HRQoL was assessed with the generic EQ-5D 3L questionnaire, developed by the EuroQol Group [38]. In this questionnaire, HRQoL is scored in five single-item dimensions: Mobility, Self-Care, Usual Activities, Pain/Discomfort and Anxiety/Depression. The subitem scores can be converted to a global health index value. This EQ-5D 3L index value has a range from − 0.594 to one, where one equals perfect health, zero equals death, and negative values are considered worse than death. The questionnaire is validated in the Norwegian population [22], and the EQ-5D 3L index value was recently shown to be responsive in glioma patients who deteriorate functionally after surgery [30].

### Ethical approval

All patients provided written informed consent prior to inclusion. The data collection and study protocol (REC ref. 2014/103-1) was approved by the Regional Committee for Medical Research in Central Norway.

### Missing data

Twenty-eight patients with missing HRQoL forms at one  month were included in analyses at baseline but excluded from the longitudinal analysis. Three patients died before follow-up, and for these patients, the EQ-5D 3L index value at one month was imputed as zero, as it equals death. Furthermore, we performed a selective imputation in 13 missing subdomains (0.06% of 2110 subdomains in total) in eleven different patients. Eleven of these subdomains were missing in preoperative data, and two at one month. Nine of these patients had filled in the disease-specific instrument EORTC QLQ-C30 and QLQ-BN20 [1] the same day as filling in EQ-5D 3L, and for these patients, we imputed a level of subdomain of EQ-5D 3L equal to the answers given in the EORTC QLQ-C30 and QLQ-BN20. In the two remaining patients, we imputed the median score from their four other subdomains of EQ-5D 3L.

### Statistical analysis

Statistical analyses were performed using SPSS version 25.0 (IBM Corporation, Armonk, New York). Statistical level of significance was set to *p* < 0.05. All tests were two-sided. Normal distribution of data was assessed with Q-Q-plots and the Shapiro-Wilk test. Central tendencies are presented as median and range for skewed continuous data. Comparisons between recurrent surgeries and primary resections for continuous skewed data were performed with the Mann-Whitney *U* test. Pearson’s chi square test or Fisher’s exact test was used for nominal and ordinal variables in contingency tables.

The EQ-5D index value was calculated according to the EuroQol scoring manual, using an empirically derived set of calculations [7]. We assessed change in EQ-5D index value from baseline to one month postoperatively by dividing it into the three categories, “improved”, “unchanged” and “deteriorated”, defined as a change greater than the minimal clinical important difference (MCID). MCID is defined as “the smallest difference in score in the domain of interest that patients perceive as important, either beneficial or harmful, and which would lead the clinician to consider a change in the patient’s management” [5] and was shown to be 0.13–0.15 for EQ-5D 3L index value in glioma patients undergoing surgery [30]. In the present study, MCID in EQ-5D 3L index value was defined as ≥ 0.15.

Possible predictors of clinically significant postoperative deteriorations (≥ 0.15 negative change in EQ-5D 3L index value) at one month with *p* < 0.1 in univariable analyses were planned to be tested for independent significance (*p* < 0.05) in a multivariable model. The following variables were screened for trends (*p* < 0.1) in univariable analyses for possible inclusion in multivariable analyses: age (y/n), preoperative KPS ≥ 70 (y/n), CCI ≥ 2 (y/n), preoperative tumour volume (ml), eloquent tumour location (y/n), gross total resection (y/n), postoperative motor or language deficits (y/n) and moderate/severe complications (defined as Landriel grade ≥ 2) (y/n).

The presence of a ceiling effect for EQ-5D 3L index values was assessed by describing the proportion of patients with the highest achievable index value. As suggested by McHorney et al. [19], the ceiling effect was considered small if ≤ 15% of the patients achieved an index value of 1.0 (maximum score) and moderate if > 15%.

Survival was assessed from date of surgery (either primary or recurrent surgeries) and stratified based on clinically significant change in EQ-5D 3L index value (improvement, unchanged and deterioration), as well as extent of surgical resection (complete radiological resection, near-total extent of resection and subtotal extent of resection). Survival analyses are presented as Kaplan-Meier plots and compared with log-rank tests. In the recurrent surgery group, 11 cases with multiple reoperations were excluded from the survival analysis, in order to not count each patient more than once.

## Results

### Patient characteristics at baseline

Baseline characteristics for preoperative data in 225 included cases are summarized in Table 1. As seen, most variables at baseline were quite balanced between recurrent surgeries and primary resections, including sex, comorbidity, neurological functional level and tumour eloquence. However, median age at surgery was significantly lower in the recurrent surgeries (59 vs. 62, *p* = 0.005), and median preoperative tumour volume was lower (14.9 vs. 25.3 ml, *p* = 0.001). Furthermore, patients undergoing recurrent resections had significantly less preoperative headache, motor deficits and cognitive deficits than patients undergoing primary surgery.

**Table 1:** Patient characteristics at baseline

Variable	Recurrent surgeries (*n* = 65)	Primary resections (*n* = 160)	*p* value
Age, median (range)	59 (28–78)	62 (29–81)	0.005
Gender, *n* (%)			0.764
Female	27 (41.5)	62 (38.8)	
Male	38 (58.5)	98 (61.3)	
Charlson Comorbidity Index ≥ 2, *n* (%)	3 (4.6)	13 (8.1)	0.567
Karnofsky performance status, *n* (%)			0.845
≥ 70	55 (84.6)	132 (82.5)	
< 70	10 (15.4)	28 (17.5)	
Preoperative symptoms, *n* (%)			
Headache	8 (12.3)	63 (39.4)	<0.001
Motor deficits	10 (15.4)	50 (31.3)	0.019
Cognitive deficits	18 (27.7)	69 (43.1)	0.035
Seizures	17 (26.2)	50 (31.3)	0.521
Preoperative tumour volume in ml, median (range)	14.91 (0.98–101.18)	25.33 (1.34–143.15)	0.001
Tumour eloquence (Sawaya), *n* (%)			0.982
Not eloquent	18 (27.7)	46 (28.7)	
Near eloquent	17 (26.2)	40 (25.0)	
Eloquent	30 (46.2)	74 (46.3)	
Time since primary resection in months, median (range)	15.8 (5.5–108.1)	N/A	N/A
Recurrence diagnosed due to, *n* (%)		N/A	N/A
New/worsened symptoms	17 (26.2)		
Routine imaging	48 (73.8)		

### Treatment results

EQ-5D 3L index values preoperatively and at one month follow up are presented in Table 2. As seen, there was no significant difference in EQ-5D 3L index values at baseline, nor at one month follow-up in the two groups. The median EQ-5D 3L index value in the recurrent surgery group was 0.796 (0.040–1.00) preoperatively and 0.760 (− 0.170 to 1.00) postoperatively and 0.760 (− 0.480 to 1.00) and 0.796 (−0.240 to 1.00), respectively, in the primary resection group. However, the median perioperative change in EQ-5D 3L index value was significantly lower for reoperations, compared with primary resections (− 0.037 vs. 0.00, *p* = 0.04). Furthermore, there was no statistically significant difference regarding complication rates, new/worsened neurological deficits, nor extent of surgical resection comparing recurrent surgeries and primary resections.

**Table 2:** Treatment results

Variables	Recurrent surgeries (*n* = 65)	Primary resections (*n* = 160)	*p* value
Postoperative complications, *n* (%)			0.266
Landriel grade I	16 (24.6)	39 (24.4)	
Landriel grade II	9 (13.8)	9 (5.6)	
Landriel grade III	2 (3.1)	4 (2.5)	
Landriel grade IV	1 (1.5)	2 (1.3)	
New/worsened language or motor deficits at discharge, *n* (%)	8 (12.3)	29 (18.1)	0.327
Extent of surgical resection, *n* (%)			0.236
Gross total resection (100%)	24 (36.9)	52 (32.5)	
Near-total resection (90–99%)	28 (27.7)	63 (39.4)	
Subtotal resection (< 90%)	23 (35.4)	45 (28.1)	
Preoperative EQ-5D index value, median (range)	0.796 (0.040–1.00)	0.760 (− 0.480 to 1.00)	0.167
Postoperative (one month) EQ-5D index value^a^, median (range)	0.760 (− 0.170 to 1.00)	0.796 (− 0.240 to 1.00)	0.352
Perioperative change in EQ-5D index value^a^, median (range)	− 0.037 (− 0.820 to 0.510)	0.000 (− 0.88 to 1.17)	0.041
Perioperative change in EQ-5D index value (MCID ≥ 0.15)^a^, *n* (%)			0.235
Improved	9 (16.4)	38 (26.8)	
Unchanged	26 (47.3)	65 (45.8)	
Deteriorated	20 (36.4)	39 (27.5)	
Survival in months^b^, median (95% CI)			
From “current” surgery	8.5 (7.2–9.9)	14.5 (12.6–16.3)	< 0.001
From primary resection	22.7 (14.8–30.7)	14.5 (12.6–16.3)	
Missing data, *n* (%)			
Lost to follow-up^c^	10 (15.4)	18 (11.3)	0.383

^a^28 cases excluded from longitudinal health-related quality of life (HRQoL) analysis due to missing HRQoL data

^b^11 cases excluded from survival analysis in order to only count each patient once

^c^Not including three patients dead before 1 month follow up, as they were included in HRQoL analyses

Table 2 shows that amidst the 55 recurrent surgeries, 20 (36.4%) reported clinically significant deterioration in HRQoL at one month, compared with 39 (27.5%) in the primary resection group. Clinically significant improvement was seen in nine cases (16.4%) vs. 38 cases (26.8%). In terms of clinically significant change in HRQoL following surgery, there was no statistically significant difference between the two groups. We were not able to identify predictors of clinically significant postoperative deterioration in EQ-5D 3L index value in the reoperation group, as none of the listed variables showed statistical trends (*p* < 0.1) in univariable analyses.

The highest achievable EQ-5D 3L index value preoperatively was seen in 14 (25.5%) recurrent resections and 38 (26.8%) primary resections, resulting in a ceiling effect considered as moderate (> 15%). All analyses were also repeated on data where patients in the recurrent resection group were only included once (first recurrent resection), but this did not alter the results in any significant way (data not shown).

### Survival analysis

**Fig. 2:**
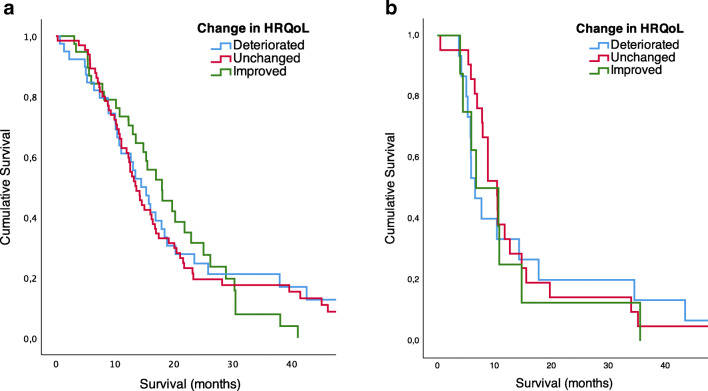
Kaplan-Meier curve of survival (log-rank test) in relation to perioperative change in health-related quality of life (HRQoL), MCID ≥ 0.15. **a** Primary resections (*n* = 142, *p* = 0.801). **b** Reoperations (*n* = 44, *p* = 0.881)

**Fig. 3:**
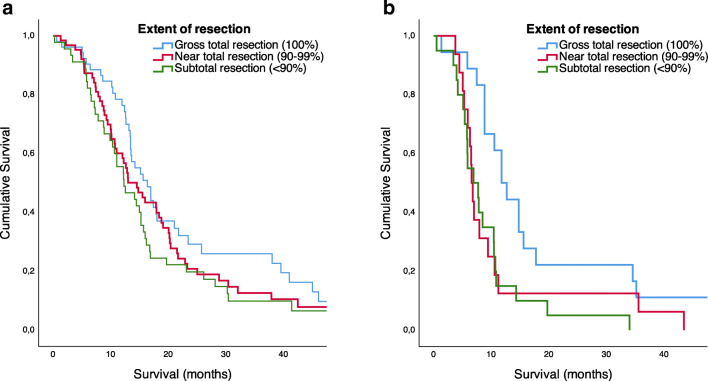
Kaplan-Meier curve of survival (log-rank test) in relation to extent of resection. **a** Primary resections (*n* = 160, *p* = 0.104). **b** Reoperations (*n* = 54, *p* = 0.015)

At the time of analysis, 23 patients were still alive, 22 of them having undergone primary resections only. Counted from the date of surgery (being primary or recurrent operations), median survival in months (95% CI) was 14.5 (12.6–16.3) and 8.5 (7.2–9.9), log-rank *p* < 0.001, respectively. When stratified for clinically significant change in EQ-5D 3L, the survival distributions were not statistically significantly different in the recurrent resection group (*p* = 0.881) nor the primary resection group (*p* = 0.801). Figure 2 shows Kaplan-Meier curves for survival in relation to perioperative change in HRQoL for primary and recurrent resections separately. Survival after recurrent surgery was associated with extent of resection (*p* = 0.015), as shown in Figure 3. However, in primary resections, this association was not statistically significant (*p* = 0.104).

## Discussion

In this longitudinal study, we found that the risk of clinically significant perioperative deterioration in HRQoL, risk of neurological deficits, complications and extents of resection were rather comparable in primary and recurrent surgeries for GBM. About a third of the patients undergoing both primary and recurrent surgeries reported clinically significant deterioration in HRQoL one month after surgery. Median perioperative change in EQ-5D index value was slightly lower in the recurrent surgery group, but the difference is not clinically significant. We were not able to identify predictors of clinically significant deterioration in patients undergoing recurrent surgeries. Gross total resection was associated with increased survival in patients undergoing recurrent surgery. Still, median postoperative survival was about six months longer after primary surgeries compared with recurrent surgeries. Although results after surgeries for recurrent GBM will depend on case selection, risks and results may not be very different from primary resections, as observed in this cohort study from our practice. Indications for surgical resection in GBM should perhaps not necessarily be much different in primary and recurrent surgeries, as patients may benefit from resection if gross total resection is obtainable with reasonable risk.

The few previous studies that evaluate patient-reported perioperative HRQoL in recurrent GBM are characterized by small patient groups, including gliomas of lower WHO grade and focusing on other outcome measures [8, 33, 35]. Surgical indications will presumably have large effects on study findings in this setting. In the present study, patients undergoing recurrent surgeries for GBM were significantly younger and had less preoperative symptoms and smaller tumour volumes, compared with patients who underwent primary resections. This, combined with the lack of difference in EQ-5D 3L index value in primary versus recurrent resections, indicates a significant selection bias, as patients in a good preoperative state are more likely to be offered repeated resections. However, the stable EQ-5D 3L index value may also in part be explained by a possible response shift, as people tend to adapt to their situation over time, thereby changing their perceived HRQoL [12]. Furthermore, a majority of the recurrent operations in this study were done in patients diagnosed with asymptomatic progression at routine follow-ups. This reduces the potential for improvement of symptoms and thereby likely also the potential of HRQoL improvement. Due to the smaller median tumour volumes in the reoperation group, the potential for symptom relief caused by reducing mass effect may also be smaller in recurrent resections.

In concordance with several studies, we found a possible survival benefit in reoperations achieving gross total resection, compared with a lesser degree of resection [2, 18, 23, 25, 28, 29, 32, 35, 39]. The effect of gross total resection at recurrence may be even larger than for primary resections. This may be explained by the fact that other treatment options are often few and less likely to have significant benefits at the time of recurrence. However, since most of these studies are neither randomized, controlled nor prospective, differentiating the effect of resection on survival from selection bias is difficult, as it leaves the possibility that the tumours that are available for gross total resection merely attain a better prognosis. Oppenlander et al. reported that maximizing the extent of resections also increases the risk of complications following surgery [23], and some studies show an increased rate of complications in reoperations compared with primary resections [6, 21, 29]. Nevertheless, in the present study, we found similar rates for complications and new neurological deficits in primary and recurrent resections. Furthermore, two recently published articles problematize the lacking use of time-dependent analysis in survival analyses, and both conclude that when treating reoperation as a time-dependent covariate, reoperation in general does not seem to prolong the overall survival [9, 40]. Hopefully, a much-needed randomized multicenter trial on reoperation of GBM, which is currently recruiting (NCT 02394626), will give us some answers on this regard.

Median postoperative survival was only 8.5 months following surgery for recurrent disease. When exploring survival in light of clinically significant patient-reported perioperative change in HRQoL, we found that there was no significant difference in survival for the three groups (improvement, unchanged, deterioration), suggesting that perioperative change in HRQoL may have a limited impact on survival. Still, we have earlier found that early postoperative deterioration in HRQoL may be associated with shorter survival in GBM patients [11].

Even though more than a third of the reoperated patients reported a clinically significant deterioration in HRQoL one month after surgery, we failed to identify predictors of this deterioration. Deterioration may not necessarily be entirely treatment related but could be related to the disease progression or the emotional distress of living with an incurable disease. Since treatment is essentially palliative, potential survival benefits should be evaluated in light of the effect reoperations have on HRQoL. Should a possible marginal increase in survival supersede the risk of deterioration in overall HRQoL in the patients remaining life span? This question remains to be considered before making treatment decisions in individual patients. The chance of obtaining a safe gross total resection and therefore a potential survival benefit should possibly be given much weight in the lack of predictors for worsening of HRQoL.

Strengths of this study include a longitudinal design with prospectively collected HRQoL data and the use of MCID defining clinically significant postoperative changes in patient-reported HRQoL. The multifaceted EQ-5D 3L questionnaire used for collection of HRQoL data was chosen due to its simple and generic nature to improve compliance. There are also some limitations. Generally, results following both primary and recurrent surgeries for GBM will depend much on treatment indications and the surgical decisions that are made, especially when evaluating parameters like survival. The selective imputation we performed might also have contributed to this selection bias. Furthermore, possible adjuvant therapy was not taken into account in the analyses. Adjuvant treatment after recurrent surgeries is highly individualized based on previous treatment responses, molecular markers (MGMT status), previous treatment, time since first-line treatment, postoperative results after recurrence operations etc. Adjuvant therapy like second-line chemotherapy, re-radiation and/or gamma knife radiosurgery might have had an impact on survival for some of the patients. We therefore cannot exclude that possible adjuvant therapy influenced outcome, neither can we rule out that this might appear as a causality because patients with prolonged survival live long enough to receive more adjuvant therapy. Another major limitation is the relatively small sample size, though exceeding other studies on the same subject. The moderate ceiling effect of the EQ-5D index value might have led to a reduced responsiveness for improvement in our study, since patients in a good preoperative condition could only stay unchanged or worsen. We chose to use patients undergoing primary surgery for GBM as a reference, in order to enhance interpretation of results. This comparison may be affected by the aforementioned selection bias as well as a possible response shift from HRQoL state at primary resection to state at re-resection [12]. Additionally, focusing on operations and not individual patients, some patients are counted more than once, either as having undergone both primary and recurrent resections or multiple resections in the study period.

## Conclusions

In this prospective cohort study comparing primary and recurrent surgeries for GBM, pre- and postoperative global HRQoL, the risk of neurological deficits, complication rates and extents of resection were rather comparable. In both groups, about a third reported clinically significant deterioration and almost half of the patients reported unchanged HRQoL perioperatively. Gross total resection was associated with increased survival in patients undergoing recurrent surgery. As surgery may prolong life in patients where gross total resection is obtainable with reasonable risk, the indication for surgical resection in GBM should perhaps not differ that much in primary and recurrent surgeries.

## Abbreviations and acronyms

CCI: Charlson comorbidity index
CI: Confidence interval
CT: Computer tomography
FLAIR: Fluid-attenuated inversion recovery
GBM: Glioblastoma
HRQoL: Health-related quality of life
KPS: Karnofsky performance status
MCID: Minimal clinically important difference
MRI: Magnetic resonance imaging
WHO: World Health Organization

## References

[1] Aaronson NK, Ahmedzai S, Bergman B, Bullinger M, Cull A, Duez NJ, Filiberti A, Flechtner H, Fleishman SB, de Haes JC (1993). The European Organization for Research and Treatment of Cancer QLQ-C30: a quality-of-life instrument for use in international clinical trials in oncology. J Natl Cancer Inst.

[2] Bloch O, Han SJ, Cha S, Sun MZ, Aghi MK, McDermott MW, Berger MS, Parsa AT (2012). Impact of extent of resection for recurrent glioblastoma on overall survival: clinical article. J Neurosurg.

[3] Brandes AA, Bartolotti M, Franceschi E (2013). Second surgery for recurrent glioblastoma: advantages and pitfalls. Expert Rev Anticancer Ther.

[4] Charlson ME, Pompei P, Ales KL, MacKenzie CR (1987). A new method of classifying prognostic comorbidity in longitudinal studies: development and validation. J Chronic Dis.

[5] Cook CE (2008). Clinimetrics corner: the minimal clinically important change score (MCID): a necessary pretense. J Man Manip Ther.

[6] De Bonis P, Fiorentino A, Anile C, Balducci M, Pompucci A, Chiesa S, Sica G, Lama G, Maira G, Mangiola A (2013). The impact of repeated surgery and adjuvant therapy on survival for patients with recurrent glioblastoma. Clin Neurol Neurosurg.

[7] Dolan P (1997). Modeling valuations for EuroQol health states. Med Care.

[8] Giovagnoli AR, Silvani A, Colombo E, Boiardi A (2005). Facets and determinants of quality of life in patients with recurrent high grade glioma. J Neurol Neurosurg Psychiatry.

[9] Goldman DA, Hovinga K, Reiner AS, Esquenazi Y, Tabar V, Panageas KS (2018). The relationship between repeat resection and overall survival in patients with glioblastoma: a time-dependent analysis. J Neurosurg.

[10] Hervey-Jumper SL, Berger MS (2014). Reoperation for recurrent high-grade glioma: a current perspective of the literature. Neurosurgery.

[11] Jakola AS, Gulati S, Weber C, Unsgard G, Solheim O (2011). Postoperative deterioration in health related quality of life as predictor for survival in patients with glioblastoma: a prospective study. PLoS One.

[12] Jakola AS, Solheim O, Gulati S, Sagberg LM (2017). Is there a response shift in generic health-related quality of life 6 months after glioma surgery?. Acta Neurochir.

[13] Kleihues P, Louis DN, Scheithauer BW, Rorke LB, Reifenberger G, Burger PC, Cavenee WK (2002). The WHO classification of tumors of the nervous system. J Neuropathol Exp Neurol.

[14] Landriel Ibanez FA, Hem S, Ajler P, Vecchi E, Ciraolo C, Baccanelli M, Tramontano R, Knezevich F, Carrizo A (2011). A new classification of complications in neurosurgery. World Neurosurg.

[15] Lasocki A, Tsui A, Tacey MA, Drummond KJ, Field KM, Gaillard F (2015). MRI grading versus histology: predicting survival of World Health Organization grade II-IV astrocytomas. AJNR Am J Neuroradiol.

[16] Louis DN, Ohgaki H, Wiestler OD, Cavenee WK, Burger PC, Jouvet A, Scheithauer BW, Kleihues P (2007). The 2007 WHO classification of tumours of the central nervous system. Acta Neuropathol.

[17] Lu VM, Jue TR, McDonald KL, Rovin RA (2018). The survival effect of repeat surgery at glioblastoma recurrence and its trend: a systematic review and meta-analysis. World Neurosurg.

[18] McGirt MJ, Chaichana KL, Gathinji M, Attenello FJ, Than K, Olivi A, Weingart JD, Brem H, Quinones-Hinojosa AR (2009). Independent association of extent of resection with survival in patients with malignant brain astrocytoma. J Neurosurg.

[19] McHorney CA, Tarlov AR (1995). Individual-patient monitoring in clinical practice: are available health status surveys adequate?. Qual Life Res.

[20] Molinari E, Mendoza TR, Gilbert MR (2018). Opportunities and challenges of incorporating clinical outcome assessments in brain tumor clinical trials. Neuro-Oncology Practice.

[21] Nieder C, Grosu AL, Molls M (2000). A comparison of treatment results for recurrent malignant gliomas. Cancer Treat Rev.

[22] Nord E (1991). EuroQol: health-related quality of life measurement. Valuations of health states by the general public in Norway. Health Policy.

[23] Oppenlander ME, Wolf AB, Snyder LA, Bina R, Wilson JR, Coons SW, Ashby LS, Brachman D, Nakaji P, Porter RW, Smith KA, Spetzler RF, Sanai N (2014). An extent of resection threshold for recurrent glioblastoma and its risk for neurological morbidity. J Neurosurg.

[24] Ostrom QT, Gittleman H, Fulop J, Liu M, Blanda R, Kromer C, Wolinsky Y, Kruchko C, Barnholtz-Sloan JS (2015). CBTRUS statistical report: primary brain and central nervous system tumors diagnosed in the United States in 2008-2012. Neuro-Oncology.

[25] Perrini P, Gambacciani C, Weiss A, Pasqualetti F, Delishaj D, Paiar F, Morganti R, Vannozzi R, Lutzemberger L (2017). Survival outcomes following repeat surgery for recurrent glioblastoma: a single-center retrospective analysis. J Neuro-Oncol.

[26] Pinsker M, Lumenta C (2001). Experiences with reoperation on recurrent glioblastoma multiforme. Zentralbl Neurochir.

[27] Prabhu VC, Barton KP, Walsh S, Borys E, Melian E (2017). Recurrent malignant gliomas: treatment options and their effect on patient’s quality of life. World Neurosurg.

[28] Quick J, Gessler F, Dutzmann S, Hattingen E, Harter PN, Weise LM, Franz K, Seifert V, Senft C (2014). Benefit of tumor resection for recurrent glioblastoma. J Neuro-Oncol.

[29] Ringel F, Pape H, Sabel M, Krex D, Bock HC, Misch M, Weyerbrock A, Westermaier T, Senft C, Schucht P, Meyer B, Simon M (2016). Clinical benefit from resection of recurrent glioblastomas: results of a multicenter study including 503 patients with recurrent glioblastomas undergoing surgical resection. Neuro-Oncology.

[30] Sagberg LM, Jakola AS, Solheim O (2014). Quality of life assessed with EQ-5D in patients undergoing glioma surgery: what is the responsiveness and minimal clinically important difference?. Qual Life Res.

[31] Sawaya R, Hammoud M, Schoppa D, Hess KR, Wu SZ, Shi WM, Wildrick DM (1998). Neurosurgical outcomes in a modern series of 400 craniotomies for treatment of parenchymal tumors. Neurosurgery.

[32] Stark AM, Hedderich J, Held-Feindt J, Mehdorn HM (2007). Glioblastoma--the consequences of advanced patient age on treatment and survival. Neurosurg Rev.

[33] Stockelmaier L, Renovanz M, Konig J, Nickel K, Hickmann AK, Mayer-Steinacker R, Nadji-Ohl M, Ganslandt O, Bullinger L, Wirtz CR, Coburger J (2017). Therapy for recurrent high-grade gliomas: results of a prospective multicenter study on health-related quality of life. World Neurosurg.

[34] Stupp R, Mason WP, van den Bent MJ, Weller M, Fisher B, Taphoorn MJ, Belanger K, Brandes AA, Marosi C, Bogdahn U, Curschmann J, Janzer RC, Ludwin SK, Gorlia T, Allgeier A, Lacombe D, Cairncross JG, Eisenhauer E, Mirimanoff RO (2005). Radiotherapy plus concomitant and adjuvant temozolomide for glioblastoma. N Engl J Med.

[35] Suchorska B, Weller M, Tabatabai G, Senft C, Hau P, Sabel MC, Herrlinger U, Ketter R, Schlegel U, Marosi C, Reifenberger G, Wick W, Tonn JC, Wirsching HG (2016). Complete resection of contrast-enhancing tumor volume is associated with improved survival in recurrent glioblastoma-results from the DIRECTOR trial. Neuro-Oncology.

[36] Sughrue ME, Sheean T, Bonney PA, Maurer AJ, Teo C (2015). Aggressive repeat surgery for focally recurrent primary glioblastoma: outcomes and theoretical framework. Neurosurg Focus.

[37] Terasaki M, Ogo E, Fukushima S, Sakata K, Miyagi N, Abe T, Shigemori M (2007). Impact of combination therapy with repeat surgery and temozolomide for recurrent or progressive glioblastoma multiforme: a prospective trial. Surg Neurol.

[38] The EuroQol Group (1990). EuroQol--a new facility for the measurement of health-related quality of life. Health Policy.

[39] Yong RL, Wu T, Mihatov N, Shen MJ, Brown MA, Zaghloul KA, Park GE, Park JK (2014). Residual tumor volume and patient survival following reoperation for recurrent glioblastoma. J Neurosurg.

[40] Zhao YH, Wang ZF, Pan ZY, Peus D, Delgado-Fernandez J, Pallud J, Li ZQ (2019). A meta-analysis of survival outcomes following reoperation in recurrent glioblastoma: time to consider the timing of reoperation. Front Neurol.

